# The prevalence and severity of lower back pain in South African university rowers

**DOI:** 10.17159/2078-516X/2021/v33i1a9323

**Published:** 2021-03-25

**Authors:** L Heyneke, A Green

**Affiliations:** Department of Sport and Movement Studies, Faculty of Health Sciences, University of Johannesburg, South Africa

**Keywords:** rowing, mechanisms of injury, risk factors, sex differences

## Abstract

**Background:**

Low back pain (LBP) is a condition prevalent among rowers due to the repetitive and physically demanding nature of rowing. Information concerning LBP among university-level rowers is, however, outdated and not widely available.

**Objective:**

To determine the prevalence, severity and disabilities of LBP among university-level rowers in South Africa.

**Methods:**

An online questionnaire, including the Athlete Disability Index (ADI) Questionnaire, was distributed to nine South African university rowing clubs. One-hundred participants aged between 18 to 30 years completed the online questionnaires.

**Results:**

Eighty-seven rowers admitted to sustaining LBP either at the time of the study or previously in their university rowing career. These rowers (n=87) completed the Athlete Disability Index (ADI) Questionnaire which provided a moderate LBP disability score (ADI score: 8.1±6.0; ADI %: 24.7%±18.1). Rowers who had been rowing for a longer duration reported a higher severity of LBP (p=0.001). There was no statistically significant difference for LBP prevalence (p=0.584), or severity (p=0.445) between the sexes. A small significant correlation between age and the ADI score (r=0.25, p= 0.021) was reported. The high prevalence and moderate severity highlight the significance of LBP among university rowers.

**Conclusion:**

This study illustrates the prevalence of LBP with moderate severity among university rowers. Future research on LBP risk factors and aetiology is recommended to decrease the negative impact of this condition.

Rowing is a physically demanding sport associated with intensive and long-term training programmes.^[1]^ The primary objective of rowing is to propel the boat using an oar as a fixed lever in flat water.^[2]^ Rowing is categorised into sculling, where the rower uses two oars, or sweep, where the rower only uses one oar, either on the left or right side.^[2]^ Furthermore, rowing can be undertaken individually or in crews of two, four, or eight, with larger crews often having the addition of a coxswain.^[2]^ The rowing stroke consists of a continuous sequence of repetitive movements which is divided into four phases, namely, the catch/preparation, the drive, the finish/release, and the recovery phases.^[2]^ The spine acts as a cantilever during the rowing stroke and has the responsibility of transferring power from the legs and arms to the oar in the water.^[3,4]^

Rowers, similar to others participating in competitive sport, have the risk of acute and chronic musculoskeletal injuries.^[1,3]^ The lower back region or the lumbar spine is the most prevalent site of injury in rowers competing at all levels.^[1–3,5,6]^ There are, however, fewer studies available concerning LBP among the university population. It is important to note that university rowing varies in different countries with regards to training intensity and competitive events ^[11]^, therefore studies conducted in other countries may not relate to the South African university rowing population. Clark et al.^[1]^ investigated South African university rowers. The study focused on multiple injuries sustained throughout a single rowing season, but with a lack of emphasis on LBP, despite the lower back being reported as the most prevalent injury site.

Previous research found that the prevalence of LBP varies but is significant in university-level rowers.^[1,6,8]^ Furthermore, despite the use of different measurement tools, it has been reported that the severity of LBP in rowers is moderate.^[1,6,8]^ There are multiple self-administered LBP questionnaires available for clinical use which assist in determining its severity ^[9],^ however, the Athletes Disability Index (ADI) Questionnaire is developed specifically for athletes to determine the influence of LBP-related disabilities on sport, exercise, and daily activities.^[10,11]^

Based on what was illustrated in previous studies,^[1,6,8]^ the primary aim of this study was to determine the prevalence, severity, and debilitating effects of LBP on university-level rowers in South Africa. The secondary aim was to establish any differences regarding LBP prevalence and severity between the sexes.

## Methods

### Participants and study design

All male and female rowers aged between 18 to 30 years who were rowing for any of the nine South African university rowing clubs at the time of the study were invited to participate in this study. This quantitative cross-sectional study design included 100 rowers (55 females and 45 males; mean age: 21.3 ± 1.7 years; rowing experience: 3.7 ± 1.9 years). The total estimated student rowing population in 2017 was 280. This was based on the number of participants who attended the 2017 University Sports of South Africa (USSA) sprints national regatta. This sample size had a margin of error of 7.8% with a 95% confidence level. All participants provided their written consent and ethical approval was granted by the Faculty’s Research Ethics Committee (REC-516-2020).

### Data collection

Data were collected using a close-ended self-administered online questionnaire. Permission was obtained from rowing club presidents from the respective universities, and thereafter they were asked to distribute the link for the online questionnaire within their structure. The questionnaire consisted of two separate sections. Section One captured demographics including age, sex, rowing experience. This section further defined LBP and determined the prevalence of this condition among the participants. If LBP was reported, participants were required to complete Section Two. Section Two was the validated ADI Questionnaire consisting of twelve questions assessing different themes, namely pain, stretching exercises, strengthening exercises, technical skills, sitting, walking, sleeping, range of motion for the back, fear avoidance behaviour, recreational activities, self-care, and sexual activity.^[11]^ This study omitted the question regarding sexual activity. Each question in the ADI is scored from 0 to 3, with the sum of the scores ranging from 0 to 36. The ADI score was converted to a percentage to determine the disability level or severity of LBP for the athlete. The disability severity percentage was defined as 0–20% – minimal, 20–40% – moderate, 40–60% – severe, 60–80% – very high, and 80–100% – sports retirement.^[10]^

### Statistical analysis

The total score for each participant’s ADI Questionnaire was calculated with Microsoft Excel, using the definitions described above. Thereafter, a descriptive analysis was performed, and data were presented as mean ± standard deviation (SD). Inferential statistics were conducted to identify differences (Mann-Whitney U Test) and relationships (Spearman’s correlation) between variables. All statistical tests were performed using the Statistical Package for the Social Sciences (SPSS), version 26.0 (IBM Inc, Chicago, IL, USA) at a significance level of p<0.05. Effect sizes were calculated using Cohen’s *d* and descriptors defined by Hopkins^[12]^ were used: trivial – *d*: 0–0.2, small – *d*: 0.2–0.6, moderate – *d*: 0.6–1.2, large – *d*: 1.2–2.0, very large – *d*: 2.0–4.0, nearly perfect – *d*: 4.0–infinity.

## Results

### Demographics

A large number (n=87) of the total sample (n=100) reported either previously or currently suffering from LBP. For the further analysis of severity only these individuals (n=87; average age: 21.4 ± 1.7 years; rowing experience: 4.4 ± 1.7 years) will be discussed.

### Prevalence

Of the total estimated population, 87 rowers (31%) reported LBP either at the time of the study (n=39) or previously in their university rowing career (n=48). There was no significant difference in the age of each group (Table) 1. There was a small difference between these two groups in number of years of rowing (p=0.017) (Table 1). On average, those who reported LBP at the time of the study had rowed for a longer time than those who reported LBP previously in their university rowing career. Additionally, there were no significant differences between the females (n=48) and males (n=39) who reported LBP for age and number of years of rowing (Table 2).

### Severity

There were no significant differences in the ADI score and disability score (%) between those participants experiencing LBP at the time or the study or previously in their university rowing career (Table 1). Additionally, there were no significant differences in the ADI score and disability score (%) between females and males, indicating similar severity between the sexes (Table 2). The disability score (%) for these groups is classified as moderately severe.^[10]^

Age was significantly correlated to the ADI score (r=0.25, p= 0.021) and the disability score (%) (r=0.25, p=0.019). The length of rowing careers yielded no significant relationships with the ADI score (%) (r=0.13, p= 0.249) or disability score (%) (r=0.12, p=0.265).

## Discussion

This study aimed to establish the prevalence of LBP in South African university rowers, and to investigate the severity among those who reported LBP and its prevalence between the sexes. The results from the current study reported that LBP was prevalent in the South African university rowing population with moderate severity. However, the severity was no different between sexes or those currently and previously reporting LBP. Finally, age was correlated to the ADI score and disability percentage.

The prevalence of LBP in this study (31%) is consistent with previous studies.^[1,6,8]^ The highest prevalence of LBP was reported by those rowing for longer than five years (45%), which is in accordance with Teitz et al. ^[6]^ who found that the number of years’ rowing is associated with LBP in university-level rowers. However, the prevalence of LBP in this study (31%) is slightly higher than the 23% of South African university rowers that reported LBP prevalence in the work of Clark et al..^[1]^ The contrast of LBP prevalence between the studies can be attributed to the different study periods. That is, Clark et al.^[1]^ only studied injuries within a single rowing season, which may have led to the exclusion of previous LBP reports. The current study investigated reports of LBP throughout the rower’s university rowing career, taking into consideration both current and previous reports, which requires that some participants had to rely on memory recall, which may not be an accurate measure of LBP prevalence. Therefore, it remains unclear whether the different study durations impacted the findings. Additionally, it is also possible that the difference between the samples of the studies may influence the LBP prevalence findings. The sample presented by Clark et al.^[1]^ had an average rowing experience of 5.0 ± 3.5 years compared to the rowers from this study who had 3.7 ± 1.9 years of experience. In contrast to this study and that of Teitz et al.,^[6]^, Clark et al. ^[1]^ found that rowers with more experience had lower reports of LBP. Furthermore, the participants from the Clark et al. ^[1]^ study ranged from university-level rowers to international medal winners, compared to the exclusive university sample used in the current study. Other studies have shown that national-level rowers’ experience have higher rates of injury than international-level rowers^[13]^, with rowing experience not influencing LBP prevalence in elite-level rowers.^[5]^ These findings may be attributed to the comprehensive health management of elite athletes through resources and support.^[13]^ Amateur athletes may not have the same level of medical care readily available and therefore experience a different approach to injury compared to those athletes of a higher level.^[13]^

This study’s combined sample (n=87) reported an average ADI disability of 24.7%±18.1%, which is classified as a moderate disability.^[10]^ These findings corresponded with previous studies which similarly reported a moderate severity of LBP in university rowers.^[1,6,8]^ Also, previous studies measured the severity of LBP using different tools, namely, time lost from participation^[1,6]^ and the visual analogue scale (VAS) pain scale.^[8]^ Measuring severity may be subjective, making it challenging for healthcare professionals to monitor a condition accurately.^[9]^ Additionally, evaluating severity through metrics, such as time lost from participation, has been found to be a poor indicator of this severity. Specifically, time lost from participation does not account for large volumes of cross-training and absence from infrequent rowing competition.^[14]^ Furthermore, the VAS pain scale has been used to measure subjective characteristics ^[15]^, which may contribute to the element of bias when reporting the severity of a condition such as LBP. The use of different scales and methods to measure LBP severity in university-level rowers has presented a challenge when attempting to draw accurate comparisons between the abovementioned studies.

The findings from the current study illustrated no sex-related differences in LBP prevalence and severity. This finding is in accordance with previous studies.^[3,4,8]^ The similarities between the sexes may be attributable to consistencies seen in loads on the spine ^[3]^, and similar training variables.^[5,6]^ Additionally, minimal kinematic differences are noted between the sexes ^[16]^, however males do present a spinal flexion angle which is largely ascribed to LBP aetiology.^[3,16]^ It is suggested that the greater muscle strength and larger muscle fibres seen in males^[17]^ may result in their erector spinae muscles overcoming the kinematic difference. Although not seen in this study, it is important to highlight the fact that higher rates of LBP in females can be expected due to these athletes having the consequences of the female triad.^[18]^ The female triad can be further compounded by the non-weight-bearing nature of rowing, as weight-bearing activities are proven to increase bone mineral density.^[19]^ From the results of the current study, it remains unclear to which degree sex-specific factors, such as kinematic differences or the female triad, are attributed to LBP.

LBP has been described as a pain, discomfort or ache felt in the low back region that presents for more than one week or interrupts at least one training session or competition.^[8]^ In the current study two groups of pain sufferers were categorised and compared. The groups were established based on current or previous experience of LBP. The results indicated that those currently experiencing LBP were not statistically different in terms of age or pain severity scores found in other studies. However, those rowers currently experiencing LBP had a slightly greater rowing experience. As previously reported ^[2]^, LBP is a chronic injury with the primary aetiology of repetitive spinal flexion which occurs during the rowing stroke. As such, those currently experiencing LBP may have had greater exposure to spinal flexion. However, more research investigating LBP aetiology and associated risk factors is required.

Finally, there was a weak relationship between age and LBP severity in this current study, which may suggest that the development of LBP is a function of age. Degenerative spinal changes are more prevalent with age.^[20]^ Furthermore, it can be speculated that spinal loading combined with the repetitive spinal flexion seen in the rowing stroke may accelerate the degeneration of the vertebral discs.^[20]^ This early degeneration seen in the spine of rowers, combined with the general age-related vertebral changes, may explain the demonstration of the weak relationship of LBP severity and aging.

LBP risk factors were not investigated in this study, therefore certain findings regarding the prevalence and severity of LBP could not be explored further. Another limitation is that the rowing level of each participant was not recorded. Additionally, it is important to note that the prevalence reported in this current study may be slightly inflated, due to the questionnaire being designed to determine the severity of LBP. The study’s design may have resulted in fewer unaffected rowers responding to the questionnaire. However, based on the current responses and estimated sample population, the prevalence (31%) is still high, considering the 7.8% margin of error. Furthermore, LBP may be a reoccurring injury among rowers ^[5]^, thus presenting these findings is important for healthcare professionals and coaches in order to emphasise the necessary precautions to limit LBP.

## Conclusion

In conclusion, this study illustrated the prevalence of LBP in the South African university rowing population with moderate severity. The severity was no different between the sexes or those currently and previously reporting LBP. Finally, age was correlated to the ADI score and disability percentage. This study highlights the importance for further investigation regarding LBP risk factors and potential causes, which may ultimately assist coaches and medical professionals in decreasing injury prevalence and improving the quality of rowing in the South African university population.

## Figures and Tables

**Table 1:** A comparison of age, rowing experience, Athlete Disability Index scores and percentages between university rowers (n=87) previously or currently suffering from lower back pain

	Previously experienced LBP (n=48)	Currently experiencing LBP (n=39)	p-value	Effect size (*d*)	Effect size
**Age (years)**	21.6 ± 1.8	21.3 ± 1.4	0.442	0.19	*trivial*
**Rowing experience (years)**	4.0 ± 1.8	4.8 ± 1.6	0.017*	−0.47	*small*
**ADI score**	8.1 ± 7.3	8.1 ± 3.9	0.175	0.00	*trivial*
**Disability score (%)**	24.7 ± 22.1	24.6 ± 11.7	0.196	0.01	*trivial*

Data are expressed as mean ± SD.

* indicates p< 0.05.

Effect size descriptors; trivial (d: < 0.2), small (d: 0.2–0.6).

ADI, Athlete Disability Index; LBP, lower back pain.

**Table 2:** A comparison of age, rowing experience, Athlete Disability Index scores and percentages between female and male university rowers (n=87) reporting historical or current lower back pain

	Female (n=48)	Male (n=39)	p-value	Effect size (*d*)	Effect size
**Age (years)**	21.7 ± 1.5	21.1 ± 1.8	0.131	0.36	*small*
**Rowing experience (years)**	4.3 ± 1.6	4.4 ± 1.9	0.472	−0.06	*trivial*
**ADI score**	8.3 ± 6.2	7.8 ± 5.7	0.445	0.08	*trivial*
**Disability score (%)**	25.4 ± 18.9	23.7 ± 17.3	0.669	0.09	*trivial*

Data are expressed as mean ± SD.

* indicates p< 0.05.

Effect size descriptors; trivial (d: < 0.2), small (d: 0.2–0.6).

ADI, Athlete Disability Index; LBP, lower back pain.
